# Complexity of the 5′ Untranslated Region of *EIF4A3*, a Critical Factor for Craniofacial and Neural Development

**DOI:** 10.3389/fgene.2018.00149

**Published:** 2018-04-25

**Authors:** Gabriella S. P. Hsia, Camila M. Musso, Lucas Alvizi, Luciano A. Brito, Gerson S. Kobayashi, Rita C. M. Pavanello, Mayana Zatz, Alice Gardham, Emma Wakeling, Roseli M. Zechi-Ceide, Debora Bertola, Maria Rita Passos-Bueno

**Affiliations:** ^1^Centro de Estudos do Genoma Humano e Células Tronco, Departamento de Genética e Biologia Evolutiva, Instituto de Biociências, Universidade de São Paulo, São Paulo, Brazil; ^2^North East Thames Genetics Service, Great Ormond Street Hospital, London, United Kingdom; ^3^Hospital de Reabilitação de Anomalias Craniofaciais, Universidade de São Paulo, São Paulo, Brazil; ^4^Instituto da Criança, Hospital das Clínicas da FMUSP, Universidade de São Paulo, São Paulo, Brazil

**Keywords:** acrofacial dysostosis, non-coding region, haplotype, expansion, crossing-over

## Abstract

Repeats in coding and non-coding regions have increasingly been associated with many human genetic disorders, such as Richieri-Costa-Pereira syndrome (RCPS). RCPS, mostly characterized by midline cleft mandible, Robin sequence and limb defects, is an autosomal-recessive acrofacial dysostosis mainly reported in Brazilian patients. This disorder is caused by decreased levels of *EIF4A3*, mostly due to an increased number of repeats at the *EIF4A3* 5′UTR. *EIF4A3* 5′UTR alleles are CG-rich and vary in size and organization of three types of motifs. An exclusive allelic pattern was identified among affected individuals, in which the CGCA-motif is the most prevalent, herein referred as “disease-associated CGCA-20nt motif.” The origin of the pathogenic alleles containing the disease-associated motif, as well as the functional effects of the 5′UTR motifs on *EIF4A3* expression, to date, are entirely unknown. Here, we characterized 43 different *EIF4A3* 5′UTR alleles in a cohort of 380 unaffected individuals. We identified eight heterozygous unaffected individuals harboring the disease-associated CGCA-20nt motif and our haplotype analyses indicate that there are more than one haplotype associated with RCPS. The combined analysis of number, motif organization and haplotypic diversity, as well as the observation of two apparently distinct haplotypes associated with the disease-associated CGCA-20nt motif, suggest that the RCPS alleles might have arisen from independent unequal crossing-over events between ancient alleles at least twice. Moreover, we have shown that the number and sequence of motifs in the 5′UTR region is associated with *EIF4A3* repression, which is not mediated by CpG methylation. In conclusion, this study has shown that the large number of repeats in *EIF4A3* does not represent a dynamic mutation and RCPS can arise in any population harboring alleles with the CGCA-20nt motif. We also provided further evidence that *EIF4A3* 5′UTR is a regulatory region and the size and sequence type of the repeats at 5′UTR may contribute to clinical variability in RCPS.

## Introduction

Over two-thirds of the human genome is comprised by repetitive elements (de Koning et al., 2011), which have been increasingly associated with functional regulatory roles. Consequently, a variety of human genetic disorders are caused by repeats in coding and non-coding sequences (Cummings and Zoghbi, 2000; Gatchel and Zoghbi, 2005; Mirkin, 2007; La Spada and Taylor, 2010; McMurray, 2010; DeJesus-Hernandez et al., 2011; Renton et al., 2011; Usdin et al., 2015; Haeusler et al., 2016). Most of these diseases are caused by unstable dynamic mutations that usually increase in size during meiotic divisions and have been associated with neurologic disorders (Virtaneva et al., 1997; Cummings and Zoghbi, 2000; Renton et al., 2011; Haeusler et al., 2016). However, poly-A repeats in *HOXD13*, the causative mechanism of a non-neurological condition, synpolydactyly, represents an exception, in which the most likely mechanism leading to increased poly-A tracts are errors in DNA replication (Muragaki et al., 1996; Warren, 1997; Brown and Brown, 2004).

We have shown that an increased number of repeats at 5′UTR of *EIF4A3* causes Richieri-Costa-Pereira syndrome (RCPS; OMIM #268305), a rare autosomal-recessive disorder affecting craniofacial and limb development, mainly described in Brazilian patients (Favaro et al., 2011, 2014; Bertola et al., 2017). RCPS individuals show a distinctive allelic pattern, determined not only by the larger number of repeats (>14 as compared to up to 12 repeats in controls), but also by the presence of a unique motif containing G instead of A nucleotide (the ‘disease-associated CGCA-20nt motif’) (Favaro et al., 2014). As the origin of the RCPS disease alleles remains unknown, characterizing the 5′UTR of *EIF4A3* in a populational level could give us clues on the mechanisms that originate the *EIF4A3* pathogenic alleles (e.g., meiotic instability or unequal crossing-over events), in addition to providing insights on the chance of RCPS arising in other populations.

We and others have shown that *EIF4A3* downregulation in cellular and animal models leads to defective neural crest cell migration/differentiation and neural stem cell apoptosis during embryonic development, paralleling RCPS cranioskeletal defects and microcephaly, respectively (Mao et al., 2016; Miller et al., 2017). However, the molecular mechanism responsible for *EIF4A3* downregulation remains entirely unknown.

Therefore, this work was undertaken to investigate the origin of the pathogenic alleles containing the disease-associated CGCA-20nt motif, as well as to evaluate the functional effects of the 5′UTR motifs on *EIF4A3* expression. Insights into the origin and effect of these complex alleles will contribute to a better understanding of regulatory features of 5′UTR regions and their role in craniofacial and neural development.

## Materials and Methods

### Ethics Approval Statement

The protocol was approved by the Ethics Committee of Instituto de Biociências at Universidade de São Paulo, Brazil (accession number 1.463.852). All individuals donated biological samples after providing signed informed consent.

### DNA Samples

To characterize *EIF4A3* 5′UTR, 380 DNA samples from unaffected individuals unrelated to RCPS families were selected from the biorepository of CEGH-CEL. For haplotype analysis, 13 additional samples were used, 12 are also from CEHG-CEL (four unaffected individuals without CGCA-20nt motifs and four with CGCA-20nt motifs; four Brazilian RCPS patients bearing different allelic structures) and one sample of a RCPS patient, from the United Kingdom, was sent for diagnosis purposes from North East Thames Genetics Service.

In order to evaluate the effects of the motifs on *EIF4A3* expression, six DNA samples carrying *EIF4A3* alleles with distinct number of motifs were selected from the biorepository of Centro de Estudos do Genoma Humano e Células Tronco (CEGH-CEL) and used for luciferase reporter assay.

For methylation assessment, we used DNA samples of RCPS patients (*n* = 6; homozygous for the 16 repeats allele) from Hospital de Reabilitação de Anomalias Craniofaciais da Universidade de São Paulo (HRAC-USP) and unaffected individuals (*n* = 7; homozygous for the eight repeats allele) from CEGH-CEL.

All samples were extracted from peripheral blood using the Gentra Systems Autopure LS (AutoGen) according to the manufacturer’s protocol.

### *EIF4A3* 5′UTR Characterization and Haplotype Analysis

Sanger sequencing of *EIF4A3* 5′UTR (NM_014740.3) and five flanking SNPs (rs11150824, rs2289534, rs3829612, rs10782008, and rs12943620) were performed using BigDye^®^ Terminator v3.1 Cycle Sequencing Kit (Thermo Fisher Scientific) and the ABI 3730 DNA Analyzer (Applied Biosystems). Sequences were analyzed using Sequencher 5.1 (Gene Codes Corporation) and Mixed Sequences Reader (Chang et al., 2012) software. The last one allowed us to better discriminate the alleles. In order to validate our analysis and obtain more reliability in our data, we randomly selected 30 heterozygous samples with different alleles’ structure to sequence each allele separately, using the Illustra GFX PCR DNA and Gel Band Purification kit (GE Healthcare) followed by Sanger sequencing.

To facilitate results interpretation, we referred SNPs markers according to their genomic relative position on the annotated plus strand (GRCh38/hg38): SNP1 (rs11150824) – SNP2 (rs2289534) – *EIF4A3* – SNP3 (rs3829612) – SNP4 (rs10782008) – SNP5 (rs12943620). Primers were designed using Primer-Blast^1^ (Untergasser et al., 2012) and are described in **Supplementary Table S1**. Linkage disequilibrium and haplotypes were inferred using Haploview software (Barrett et al., 2005).

### Luciferase Assay

Although the larger alleles (15 or 16 repeats) have been associated with decreased *EIF4A3* expression, the causal relationship between number and/or pattern of repeats and gene downregulation is still unknown. Therefore, we investigated the role of the 5′UTR motifs on *EIF4A3* expression by luciferase assay.

Sequences of interest of unaffected and RCPS individuals were amplified by PCR (primer sequences in **Supplementary Table S1**), purified using the Illustra GFX PCR DNA and Gel Band Purification kit (GE Healthcare) and cloned into the pGL4.24[*luc2P*/minP] vector (Promega), upstream of a minimal promoter and the *luc2P* gene. Sanger sequencing confirmed all constructs: pGL4.24 vectors carrying control alleles with 4 repeats (3 CACA-20-nt and 1 CA-18-nt), 7 repeats (4 CACA-20-nt, 1 CA-18-nt, 1 CACA-20-nt, and 1 CA-18-nt), 10 repeats (7 CACA-20-nt, 1 CA-18-nt, 1 CACA-20-nt, and 1 CA-18-nt) and 12 repeats (1 CACA-20-nt, 10 CGCA-20nt, and 1 CA-18-nt); pGL4.24 vectors carrying pathogenic alleles with 14 repeats (2 CACA-20nt, 10 CGCA-20nt, 1 CACA-20nt, and 1 CA-18nt) and 16 repeats (1 CACA-20nt, 13 CGCA-20nt, 1 CACA-20nt, and 1 CA-18nt). In order to investigate the individual effect of each motif, differing in the composition of central nucleotides, we also constructed vectors carrying sequences with only 1 CA-18-nt, 1 CACA-20-nt, and 1 CGCA-20nt, synthesized by Integrated DNA Technologies (IDT).

Human embryonic kidney (HEK) 293T cells, cultured in high glucose DMEM supplemented with 1% penicillin/streptomycin and 10% fetal bovine serum (FBS) (all provided by Life Technologies), were plated in 96-well plates 24 h prior to transfection (2 × 10^4^ cells/well). Transient transfections were performed in triplicate by using TurboFectin 8.0 (OriGene) according to the manufacturer’s instruction. Cells were cotransfected with 180 ng of the pGL4.24 constructs and 20 ng of the pRL-SV40 vector (Promega) containing the Renilla luciferase gene, used as a transfection control. The plasmid pLuc generated from pGL3-control template was used as positive control (Soltys et al., 2013). Forty-eight hours after DNA transfection, luciferase activity was measured with the Dual-Glo^®^ Luciferase Assay System in a GloMax Multi 96-microplate Luminometer (Promega). Firefly luminescence results were normalized by Renilla luminescence and the relative luciferase activity was determined. Statistical analyses were performed with one-way ANOVA and Tukey *post hoc* test. Significance was set at *p* < 0.05.

### Methylation Assay

Indeed, methylated CpGs are involved in gene repression especially when occurring at promoter/5′UTR (Feil and Fraga, 2012; Schübeler, 2015). Since *EIF4A3* 5′UTR motifs are CG-rich and the disease CGCA-20nt allele shows increased number of CpGs compared to control alleles, we evaluated DNA hypermethylation as a plausible mechanism behind *EIF4A3* downregulation in RCPS patients.

One microgram of genomic DNA from each sample were submitted to bisulfite conversion using EpiTect Bisulfite Conversion Kit (QIAgen). Bisulfite converted DNA was subsequently used for PCR, in which primers were designed with MethPrimer^2^ (Li and Dahiya, 2002) and are shown in **Supplementary Table S1**. Amplicons were checked by agarose electrophoresis and cloned using the TOPO TA Cloning Kit for sequencing (Thermo Fisher Scientific). Sanger sequencing was carried out for 10 clones per sample using the BigDye^®^ Terminator v3.1 Cycle Sequencing Kit (Thermo Fisher Scientific) and the ABI 3730 DNA Analyzer (Applied Biosystems). Sequencing files were then analyzed for methylation quantification using the online tool BISMA – Bisulfite Sequencing DNA Methylation Analysis (Rohde et al., 2010) with lower threshold conversion rate at 95%, lower threshold sequence identity at 90%, upper threshold of N-sites at cytosine positions at 20% and per threshold gaps allowed at 20% as filtering parameters. Methylation values were computed and differences between groups tested using Fisher’s Exact Test. Significance was set at *p* < 0.05.

## Results

The *EIF4A3* 5′UTR is characterized by the presence of 18- or 20-nucleotide-long motifs differing in the composition of central nucleotides, namely CA-18nt (TCGGCAGCGG**CA**GCGAGG), CACA-20nt (TCGGCAGCGG**CACA**GCGAGG), and CGCA-20nt (TCGGCAGCGG**CGCA**GCGAGG) (Favaro et al., 2014). Unaffected individuals have 3–12 repeats composed mostly by CA-18nt and CACA-20nt motifs, while RCPS patients have 14–16 repeats, with a higher number of the CGCA-20nt motif (Favaro et al., 2014). The motif CGCA-20nt will be herein referred as ‘disease-associated CGCA-20nt motif.’

### Unaffected Individuals Are Mostly Heterozygous and May Also Present CGCA-20nt Motifs

**FIGURE 1:**
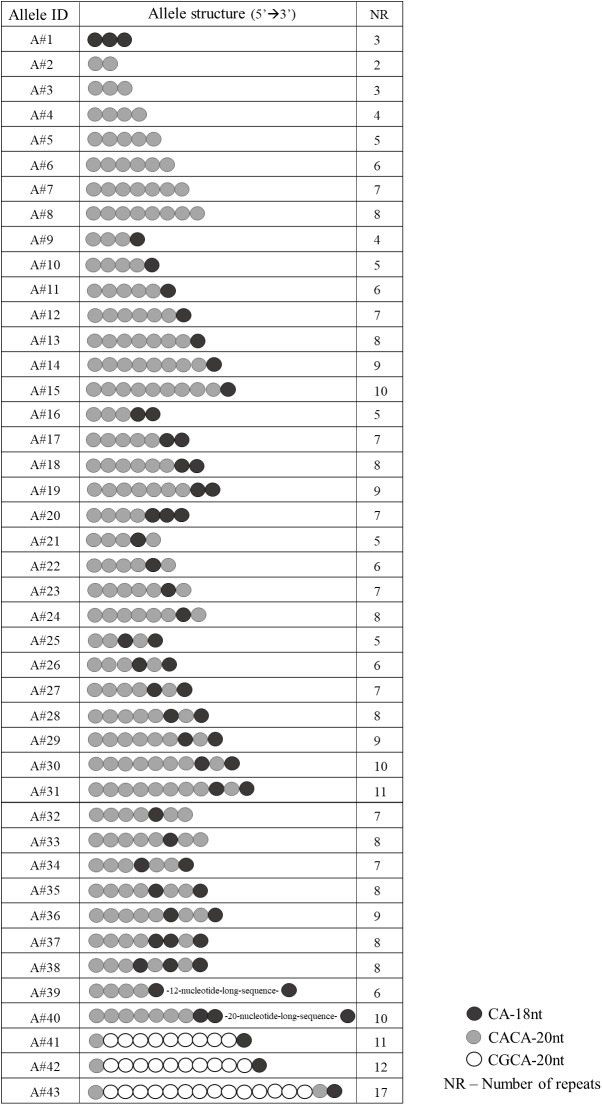
Schematic representation of the 43 allele structures identified in the 380 unaffected individuals.

Sanger sequencing of *EIF4A3* 5′UTR in 380 unaffected individuals revealed 43 different alleles (**Figure 1**) in heterozygosis in 85% of individuals. The total number of repeats per allele varied from 2 to 17, and the most common alleles contained 7 (25.46%) or 8 (23.21%) repeats (**Supplementary Figure S1**). The number and organization of the CA-18nt or CACA-20nt motifs varied between the alleles. We identified not only alleles containing a single motif, but also complex alleles with multiple organizations of the CA-18nt or CACA-20nt motifs (**Figure 1**). For example, one allele exclusively constituted by the CA-18nt motif was found. As for the other alleles, the number of the first CACA-20nt motifs varied from 2 to 8 with or without different combinations of CA-18nt motifs (**Figure 1**). The most common allele contained seven repeats and was constituted by four repeats of CACA-20nt motif followed by 1 CA-18nt, 1 CACA-20nt, and 1 final CA-18nt. We also identified two atypical alleles comprising 12- or 20-nucleotide-long sequences inserted between motifs, which do not align to any viral sequences or neighboring genes (data not shown). Finally, the disease-associated CGCA-20nt motif, originally found only among RCPS patients, was identified here in eight heterozygous control individuals, within alleles with 11 or more repeats (**Figure 1**). The largest allele (17 repeats) was found in heterozygosis in one control individual and contained 14 CGCA-20nt motifs. This allele must be pathogenic when in homozygosis.

### Haplotype Analysis Suggests That the RCPS Allele Originated More Than Once

**FIGURE 2:**
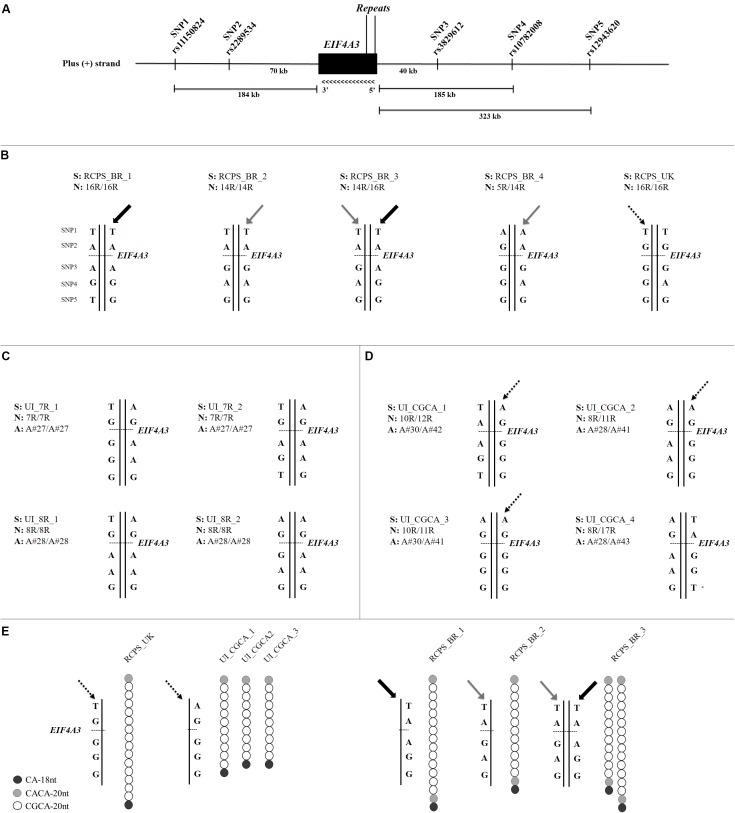
**(A)** Schematic representation of the SNPs and microsatellite markers flanking *EIF4A3* (11,970 bp). We used five SNPs for haplotype characterization and numbered them as following: SNP1 (rs11150824), SNP2 (rs2289534), SNP3 (rs3829612), SNP4 (rs10782008), and SNP5 (rs12943620), according to their relative genomic position on the annotated plus strand (GRCh38/hg38). **(B)** Haplotype analysis of five RCPS patients, four Brazilians, and one from the United Kingdom. Gray arrows indicate the haplotype associated with the 14-repeat alleles and black arrows indicate the haplotype associated with the 16-repeat alleles in Brazilian RCPS. UK RCPS presented a similar haplotype observed in the unaffected individuals with the disease CGCA-20nt motif (dotted arrow). **(C)** Haplotype analysis of four unaffected individuals without the disease CGCA-20nt motif. These individuals are homozygous for seven or eight repeats. **(D)** Haplotype analysis of four unaffected individuals with the disease CGCA-20nt motif. Dotted arrows indicate the haplotype present in three of these individuals. **(E)** Comparison between haplotypes of the UK RCPS patient, the three unaffected individuals with the CGCA-20nt motif and the three Brazilian RCPS patients. S is the sample ID, N is the total number of repeats for each allele and A is the allele structure (**Figure 1** and **Supplementary Table S2**). Each bar represents an allele.

To understand the origin of the alleles containing the disease-associated CGCA-20nt motif, we initially characterized the haplotypes of 13 samples: five affected individuals (four Brazilian and one from the United Kingdom) and eight control individuals (**Supplementary Table S2**), using five SNPs flanking *EIF4A3* and spanning 519 kb (**Figure 2A**). We observed a weak linkage disequilibrium in this block (*D*’ < 0.48).

The Brazilian RCPS patients showed at least two different haplotypes. The haplotype associated with the pathogenic 14-repeat allele is different from the one associated with the 16-repeat allele (**Figure 2B**), which in turn were not observed in any of the tested unaffected individuals (**Figure 2C**). The fact that the two affected alleles with 14 and 16 repeats are embedded within different haplotypes, undetected in unaffected individuals, suggests a distinct origin for these two different-sized pathogenic alleles in our population. Next, we analyzed the homozygous (16 repeats) UK RCPS sample, which showed a different haplotype from those observed in the Brazilian RCPS (**Figure 2B**). However, one UK RCPS haplotype is similar to a haplotype observed in three of the unaffected individuals carrying the disease-associated CGCA-20nt motif (**Figures 2B,D**). Besides, the alleles in these control individuals also show similarities in motif organization in relation to the allele in the UK RCPS patient, suggesting that these alleles possibly share a common origin (**Figure 2E** and **Supplementary Table S2**).

### Transcriptional Activity Is Inversely Correlated to Motif Number at *EIF4A3* 5′UTR

**FIGURE 3:**
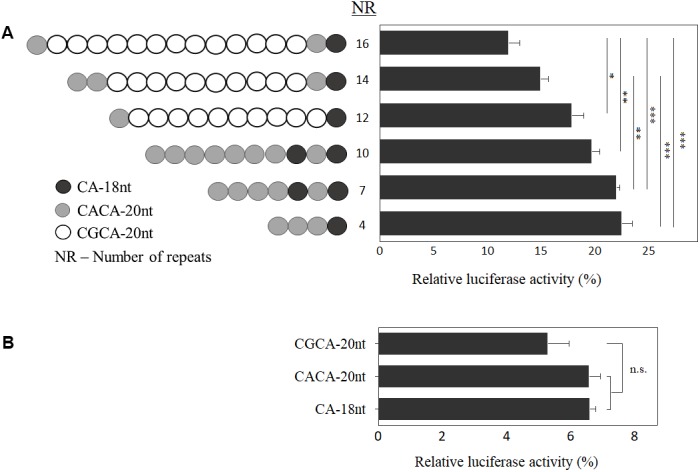
Functional analysis of *EIF4A3* 5′UTR motifs. Graph depicting the relative luciferase activity, in percentage, of the constructs carrying different number of motifs **(A)** and of each motif independently **(B)**. Data are represented as mean ± SEM of five experiments, which were performed in triplicate. Relative luciferase activity = [(experimental sample ratio) – (negative control ratio)/(positive control ratio) – (negative control ratio)]. ^∗∗∗^*p* < 0.001, ^∗∗^*p* < 0.01, ^∗^*p* < 0.05 and ns, non-significant; one-way ANOVA with Tukey *post hoc* tests.

To clarify the functional role of the allelic structure at *EIF4A3* 5′UTR, we generated constructs varying in size and composition (**Figure 3**), and carried out luciferase reporter assays. Inverse correlation between number of motifs and luciferase activity was observed (*n* = 4 independent experiments; *p* < 0.05; **Figure 3A**). Further, by investigating each motif individually, there was a discrete reduction in expression for the disease-associated CGCA-20nt, albeit not statistically significant (*n* = 3 independent experiments; **Figure 3B**).

In order to address whether the motif sequence plays a role in *EIF4A3* gene expression, based on data shown in **Figure 3A**, we calculated the effect per motif type on luciferase activity. Comparing the luciferase activity of alleles with similar composition of motifs, carrying 4 and 10 repeats (22.45% and 19.69%, respectively), we observed a decrease of 2.76% in expression, which represents a reduction of about 0.46% per CACA-20nt motif added in the allele structure. On the other hand, between the alleles carrying 12 and 16 repeats (17.8% and 11.96%, respectively), with comparable allelic structure, the difference was 5.84%, which means a reduction in luciferase activity of 1.46% per CGCA-20nt motif added (three times higher). Based on these results, we suggest that both size and allele sequence play a role in gene regulation.

### 5′UTR Hypermethylation Is Not Responsible for *EIF4A3* Downregulation

We inspected methylation levels at the 5′UTR of *EIF4A3*, as the increased number of repeats in RCPS patients leads to gain of 37 CpG sites. We observed that both RCPS and controls did not show abundant methylation of this region (1.7% and 2.8% of methylated CpGs, respectively), with a discrete reduction of methylation in RCPS (*p* < 0.05) (**Supplementary Table S3**). There was no evident methylation variation at any specific CpG between RCPS and controls, as both presented low methylation levels.

## Discussion

Expansions at non-coding regions have been extensively described in neurological disorders, and characterization of these regions have greatly contributed to the understanding of novel regulatory mechanisms (Gatchel and Zoghbi, 2005; Mirkin, 2007; La Spada and Taylor, 2010; McMurray, 2010; Russo et al., 2015; Usdin et al., 2015; Haeusler et al., 2016). Despite the great advances in genome sequence analysis, DNA of repetitive regions is still difficult to be sequenced. In fact, the *EIF4A3* 5′UTR is not covered in GnomAD database, which reinforces the importance of characterizing this region through Sanger sequencing.

In this study, Sanger sequencing analysis of *EIF4A3* 5′UTR in 380 unaffected individuals revealed 43 different alleles, with the most common alleles containing seven or eight repeats. Some of these alleles presented only one type of motif (CA-18nt or CACA-20nt) with different total number of repeats, while others presented a visible combination of these two common motifs, suggesting that these alleles may have originated through unequal crossing-over events. These results show its polymorphic nature and confirm the structural complexity, and uniqueness of this region, which was not comparable to any gene in which dynamic pathogenic expansions at non-coding regions had been reported (Brook et al., 1992; Campuzano et al., 1996; Moseley et al., 2006; Daughters et al., 2009; Galloway and Nelson, 2009; Sato et al., 2009; DeJesus-Hernandez et al., 2011; Kobayashi et al., 2011). In this enlarged cohort, 1% of the alleles (8/760) harbors the disease-associated CGCA-20nt motif. Interestingly, the largest alleles in this cohort (≥11 repeats), including one with 17 repeats, contained the disease CGCA-20nt motif. These results suggest that RCPS could occur in any population containing alleles with the CGCA-20nt motif. Indeed, one of the patients here included is from United Kingdom (Bertola et al., 2017).

The haplotype analyses were performed in order to get insights on the origin of the pathogenic alleles with increased number of repeats. Results revealed that the pathogenic alleles with 14 and 16 repeats of Brazilian patients have distinct origins, which in turn are different from the haplotypes of the UK RCPS patient. These results suggest that the pathogenic alleles have arisen more than once. It is of note that the UK RCPS patient shares a common haplotype and also a similar motif structure with three unaffected individuals carrying the disease-associated CGCA-20nt motif, suggesting a common ancestral among them. It is possible that, similarly to the alleles in the control population, the affected alleles may have arisen also through unequal crossing. This hypothesis is also supported by the observation that the number of repeats at *EIF4A3* 5′UTR seems to be stable across generations (Favaro et al., 2014). This phenomenon is more comparable to the one observed in synpolydactyly, in which the poly-A at the 3′end of *HOXD13* might have originated by unequal crossing over as it is quite stable when transmitted across generations (Warren, 1997), as opposed to dynamic mutations observed in neurological conditions, which have arisen only once (Virtaneva et al., 1997; Gatchel and Zoghbi, 2005; Mirkin, 2007; La Spada and Taylor, 2010; McMurray, 2010; DeJesus-Hernandez et al., 2011; Renton et al., 2011; Usdin et al., 2015; Haeusler et al., 2016).

Next, we demonstrated that the number and allele sequence of motifs at 5′UTR is involved in *EIF4A3* expression. These results thus suggest a potential *cis*-acting regulatory mechanism for these motifs on gene expression and confirm our previous finding that the affected alleles were associated with *EIF4A3* downregulation in cells from different tissues from RCPS patients, including peripheral blood (Favaro et al., 2014; Miller et al., 2017). DNA hypermethylation is not the mechanism for *EIF4A3* downregulation in RCPS patients, as RCPS and control blood samples showed similar methylation levels. Hypomethylation, as observed at *EIF4A3* 5′UTR, is consistent with transcriptional activity (Baubec and Schübeler, 2014; Schübeler, 2015). There could be alternative mechanisms by which the increased number of motifs could repress *EIF4A3* expression: the disease CGCA-20nt could create binding sites for repressor proteins and act as a silencer; or more complex mechanisms, including post-translational events, could be involved. Further functional studies are needed to pinpoint the exact molecular mechanism underlying *EIF4A3* downregulation.

The inverse correlation between number of repeats and *EIF4A3* expression levels suggests that the structure of the 5′UTR *EIF4A3* may modulate the phenotype. Indeed, a broader phenotypic spectrum within RCPS has been observed, ranging from individuals with severe phenotype (homozygous for the 16-repeat allele or heterozygous for the 15- and 16-repeat alleles) to individuals with less severe skeletal involvements, harboring smaller number of motifs (homozygous for the 14-repeat allele or compound heterozygous for 14 repeats and a point mutation (c.809A>G) (Favaro et al., 2014; Bertola et al., 2017). Despite limitations in sample size, these results suggest that RCPS phenotypic variability depends upon the number and allele sequence of repeats at the 5′UTR of *EIF4A3*.

## Conclusion

In summary, we provide evidence supporting that the *EIF4A3* 5′UTR is highly polymorphic, comprising at least 43 different alleles, which may have originated through extensive recombination in this region. Haplotype analysis in control and in affected individuals suggests that there are more than one haplotype associated with the disease and RCPS alleles might have originated from unequal crossing-over events. We also provided further evidence that *EIF4A3* 5′UTR is a regulatory region, and that *EIF4A3* downregulation in RCPS is not mediated by CpG methylation. Moreover, our findings provide insights to explain clinical variability in RCPS.

## Author Contributions

DB, RP, and MZ provided the Brazilian DNA samples. AG and EW provided DNA sample and clinical information from the UK RCPS patient. GH, CM, and MP-B conceived and designed the study. GH performed the *EIF4A3* 5′UTR characterization and haplotype analyses. LB assisted with haplotype and linkage disequilibrium analyses. CM carried out the luciferase assay and LA performed the methylation assay. GH, CM, LA, LB, GK, and MP-B discussed and interpreted the results and wrote the manuscript. GH and CM have a major contribution in writing the manuscript. All authors contributed to the final version and approved the manuscript.

## Conflict of Interest Statement

The authors declare that the research was conducted in the absence of any commercial or financial relationships that could be construed as a potential conflict of interest.

## Footnotes

1 https://www.ncbi.nlm.nih.gov/tools/primer-blast/

2 www.urogene.org/methprimer/

## Supplementary Material

FIGURE S1 Distribution of number of alleles per total number of repeats identified in the 380 samples of unaffected individuals. This analysis did not take in consideration the motifs’ structure.

TABLE S1 Primer sequences.

TABLE S2 List of the 13 samples used in haplotype analysis and the description of motifs’ structure reported in each allele.

TABLE S3 Methylation levels in the analyzed regions in RCPS patients and control individuals; *p* = 0.0461, Fisher’s Exact Test.
