# Supplementary material for: Complexity of the 5′ Untranslated Region of EIF4A3, a Critical Factor for Craniofacial and Neural Development
Source: Front Genet. 2018 Apr 25;9:149. doi: 10.3389/fgene.2018.00149 (PMC5996909; doi:10.3389/fgene.2018.00149)
Supplement: TABLE S1 — Primer sequences. [file Table_1.PDF]

Target	Forward strand (5' → 3')	Reverse strand (5' → 3')
EIF4A3<br>Luciferase assay	AAAGGTACCACGCCCAGTTCCCTTTCAC	AAAAAGCTTGAACGTGGGGGTCACATC
EIF4A3<br>Methylation analysis	GYGAGAGTAGAAATATTTTATTTTTT	TCATATCTTCCTCTTTAAACAACC
EIF4A3<br>Characterization analysis	ACGCCCAGTTCCCTTTCAC	GAACGTGGGGGTCACATC
rs11150824	TGAGCAAAATATGGCAGGAAGTC	AGAATGCCCAGCGGTTGAC
rs2289534	ACAGAGGGTGCACCTTGATCC	AAAGGGACACCTTTACCTCCC
rs3829612	GAAGTCTGCACGTCGGGGT	AGTCTCATGTTTTCCGGTTCCC
rs10782008	TGACTTCTGACACTTTCTTTTCGC	AGCTGGAAGATGTGGCTGTC
rs12943620	GACGCACAAAACAGATCATCGC	ACATCTGGGACCGCAAGCTG
