# Supplementary material for: Complexity of the 5′ Untranslated Region of EIF4A3, a Critical Factor for Craniofacial and Neural Development
Source: Front Genet. 2018 Apr 25;9:149. doi: 10.3389/fgene.2018.00149 (PMC5996909; doi:10.3389/fgene.2018.00149)
Supplement: TABLE S2 — List of the 13 samples used in haplotype analysis and the description of motifs’ structure reported in each allele. [file Table_2.PDF]

Sample ID	Sample description	Number of repeats (R)	Alleles' structure	
UI_7R_1	Unaffected individual	7R/7R	A#27	A#27
UI_7R_2	Unaffected Individual	7R/7R	A#27	A#27
UI_8R_1	Unaffected Individual	8R/8R	A#28	A#28
UI_8R_2	Unaffected Individual	8R/8R	A#28	A#28
UI_CGCA_1	Unaffected Individual with CGCA-20nt	10R/12R	A#30	A#42
UI_CGCA_2	Unaffected Individual with CGCA-20nt	8R/11R	A#28	A#41
UI_CGCA_3	Unaffected Individual with CGCA-20nt	10R/11R	A#30	A#41
UI_CGCA_4	Unaffected Individual with CGCA-20nt	8R/17R	A#28	A#43
RCPS_BR_1	RCPS Brazilian	16R/16R	1 CACA-20nt + 13 CGCA-20nt + 1 CACA-20nt + 1 CA-18nt	1 CACA-20nt + 13 CGCA-20nt + 1 CACA-20nt + 1 CA-18nt
RCPS_BR_2	RCPS Brazilian	14R/14R	1 CACA-20nt + 11 CGCA-20nt + 1 CACA-20nt + 1 CA-18nt	1 CACA-20nt + 11 CGCA-20nt + 1 CACA-20nt + 1 CA-18nt
RCPS_BR_3	RCPS Brazilian	14R/16R	1 CACA-20nt + 11 CGCA-20nt + 1 CACA-20nt + 1 CA-18nt	1 CACA-20nt + 13 CGCA-20nt + 1 CACA-20nt + 1 CA-18nt
RCPS_BR_4	RCPS Brazilian	5R/14R	4 CACA-20nt + 1 CA-18nt	2 CACA-20nt + 2 CGCA-20nt + 1 CACA-20nt + 3 CGCA-20nt + 1 CACA-20nt + 3 CGCA-20nt + 2 CACA-20nt
RCPS_UK	RCPS London	16R/16R	1 CACA-20nt + 14 CGCA-20nt + 1 CA-18nt	1 CACA-20nt + 14 CGCA-20nt + 1 CA-18nt
