# Supplementary material for: Complexity of the 5′ Untranslated Region of EIF4A3, a Critical Factor for Craniofacial and Neural Development
Source: Front Genet. 2018 Apr 25;9:149. doi: 10.3389/fgene.2018.00149 (PMC5996909; doi:10.3389/fgene.2018.00149)
Supplement: TABLE S3 — Methylation levels in the analyzed regions in RCPS patients and control individuals; p = 0.0461, Fisher’s Exact Test. [file Table_3.pdf]

	RCPS		Controls	
	Number of CpGs	%	Number of CpGs	%
Methylated CpGs	40	1.7	29	2.8
Non-methylated CpGs	2352	98.3	1011	97.2
Total	2392		1040	
